# Incidence of catastrophic health spending in Indonesia: insights from a Household Panel Study 2018–2019

**DOI:** 10.1186/s12939-023-01980-w

**Published:** 2023-09-06

**Authors:** Rifqi Abdul Fattah, Qinglu Cheng, Hasbullah Thabrany, Dwidjo Susilo, Aryana Satrya, Manon Haemmerli, Soewarta Kosen, Danty Novitasari, Gemala Chairunnisa Puteri, Eviati Adawiyah, Andrew Hayen, Lucy Gilson, Anne Mills, Viroj Tangcharoensathien, Stephen Jan, Augustine Asante, Virginia Wiseman

**Affiliations:** 1 Centre for Social Security Studies, University of Indonesia, Jakarta, Indonesia; 2 Kirby Institute, University of New South Wales, Sydney, Australia; 3 Kirby Institute, UNSW Australia, Level 6, Wallace Wurth Building, High Street, 2052 Kensington, NSW Australia; 4 ThinkWell Indonesia, Jakarta, Indonesia; 5 Faculty of Medicine and Health, University of Muhammadiyah, Jakarta, Indonesia; 6 Department of Management, Faculty of Economics, University of Indonesia, Depok, Indonesia; 7 Department of Global Health and Development, London School of Hygiene & Tropical Medicine, London, UK; 8 Independent Consultant, Jakarta, Indonesia; 9 Centre for Health Economics and Policy Studies, Faculty of Public Health, University of Indonesia, Jakarta, Indonesia; 10 Biostatistics and Demography Department, Faculty of Public Health, University of Indonesia, Jakarta, Indonesia; 11 School of Public Health, University of Technology Sydney, Sydney, Australia; 12 Health Policy and Systems Division, School of Public Health, University of Cape Town, Cape Town, South Africa; 13 International Health Policy Programme, Ministry of Public Health, Nonthaburi, Thailand; 14 The George Institute for Global Health, University of New South Wales, Sydney, Australia; 15 Faculty of Medicine and Health, School of Public Health, The University of Sydney, Sydney, Australia; 16 School of Public Health & Community Medicine, University of New South Wales, Sydney, Australia

**Keywords:** Catastrophic health spending, Indonesia, Jaminan Kesehatan Nasional, Out-of-pocket payment

## Abstract

**Background:**

Indonesia implemented one of the world’s largest single-payer national health insurance schemes (the Jaminan Kesehatan Nasional or JKN) in 2014. This study aims to assess the incidence of catastrophic health spending (CHS) and its determinants and trends between 2018 and 2019 by which time JKN enrolment coverage exceeded 80%.

**Methods:**

This study analysed data collected from a two-round cross-sectional household survey conducted in ten provinces of Indonesia in February–April 2018 and August–October 2019. The incidence of CHS was defined as the proportion of households with out-of-pocket (OOP) health spending exceeding 10% of household consumption expenditure. Chi-squared tests were used to compare the incidences of CHS across subgroups for each household characteristic. Logistic regression models were used to investigate factors associated with incurring CHS and the trend over time. Sensitivity analyses assessing the incidence of CHS based on a higher threshold of 25% of total household expenditure were conducted.

**Results:**

The overall incidence of CHS at the 10% threshold fell from 7.9% to 2018 to 4.4% in 2019. The logistic regression models showed that households with JKN membership experienced significantly lower incidence of CHS compared to households without insurance coverage in both years. The poorest households were more likely to incur CHS compared to households in other wealth quintiles. Other predictors of incurring CHS included living in rural areas and visiting private health facilities.

**Conclusions:**

This study demonstrated that the overall incidence of CHS decreased in Indonesia between 2018 and 2019. OOP payments for health care and the risk of CHS still loom high among JKN members and among the lowest income households. More needs to be done to further contain OOP payments and further research is needed to investigate whether CHS pushes households below the poverty line.

**Supplementary Information:**

The online version contains supplementary material available at 10.1186/s12939-023-01980-w.

## Background

Achieving universal health coverage (UHC) is one of the overarching targets of the 2030 sustainable development goals (SDGs) [1]. In addition to improved access to health services, financial risk protection is another UHC goal, which is defined as access to all needed quality health services without financial hardship [2]. In September 2019, the United Nations (UN) General Assembly held a high-level meeting, where all UN Member States reaffirmed their commitment to UHC [3]. Despite this commitment, the burden imposed by out-of-pocket (OOP) health payments still results in financial hardship for millions of people who seek health care globally [4]. It is estimated that globally approximately 70 million people were pushed into extreme poverty and a further 435 million people were pushed deeper into extreme poverty in 2017 by OOP health payments [5]. While OOP payments have been steadily declining in Indonesia over the past 10 years, National Health Accounts (NHA) data show that in 2020, OOP payments accounted for around one-third of total current health expenditure [6]. This rate was higher than that of Singapore (29%), Thailand (10%) and Brunei Darussalam (5%) [7].

The level of financial protection is measured by catastrophic health spending (CHS) which is defined under SDG indicator 3.8.2 as OOP health payments greater than 10% or 25% of total household expenditure [1]. Studies have found that CHS reduces the consumption of essential commodities such as food, housing, and education of children [8], pushing many families into poverty [9]. In 2017, Wagstaff et al. demonstrated that the global incidence of CHS at the 10% threshold (the most commonly used threshold for global monitoring of CHS) had increased from 9.7% to 2000, to 11.4% in 2005, and to 11.7% in 2010 [10]. Wagstaff et al. also reported that the expansion of health insurance coverage in Indonesia did not coincide with a reduction in the incidence of CHS between 2000 and 2010 [10]. A more recent study by the World Health Organization (WHO) and the World Bank showed that the incidence of CHS in Indonesia had worsened over time, rising from 3.6% to 2015 to 4.5% in 2017 at the 10% threshold [5, 11].

In 2014, the Government of Indonesia introduced its national health insurance program *Jaminan Kesehatan Nasional* (JKN) managed by the Social Security Organizing Agency for Health (*Badan Penyelenggara Jaminan Kesehatan-BPJS Kesehatan*) in order to provide universal access to health services with a focus on targeting and subsidizing care for the poor. The JKN mandates all wage earners (formal sector employees) to contribute 1% of their payroll to the JKN with employers required to provide matching funding of an additional 4% of their employees’ wages. The poorest 40% of the population (who are deemed incapable of contributing) are fully subsidized by the central, provincial, city or district governments. Non-wage earners pay a fixed contribution, at three different levels, Indonesian Rupiah (IDR) 42,000, 100,000 and 150,000 per person per month, based on the choice of ward class selected by the person [12]. The JKN covers comprehensive medical care defined as medically necessary health care. Public and private primary health care providers are paid under a capitation model, while public and private hospitals are paid using the Indonesian Case Base Groups (INA-CBG) for all outpatient and inpatient care [13]. In theory, when a JKN member attends a facility within the JKN network, no co-payment or cost-sharing is required. By December 2022, the JKN Administrator reported covering 90% of the Indonesian population [14].

Although the WHO and the World Bank regularly publish data on the incidence of CHS globally, including for Indonesia [5, 15], they have used national socioeconomic survey data that were not originally designed for measuring CHS and consequently do not capture all major aspects of OOP spending on health care in Indonesia. Moreover, the incidence of CHS from 2018 onward has not been published. This study is a retrospective data analysis of a two-round household survey that collected comprehensive information on OOP health spending in Indonesia between 2018 and 2019. The aim of this study was to assess the incidence, determinants, and trend of CHS in the JKN era between 2018 and 2019 by which time JKN enrolment coverage exceeded 80%. This updated evidence will help the Indonesian government design policy reforms to address current gaps in financial protection.

## Methods

### Study setting

Indonesia is the largest economy in Southeast Asia, and a member of the Group of Twenty (G20), a group of finance ministers and central bank governors from 19 of the world’s largest economies and the European Union. Due to the economic impact of the pandemic, Indonesia went from upper-middle income to lower-middle income status in July 2021, but regained upper middle-income country status in 2023. It is the world’s largest archipelago and the fourth most populous country in the world with approximately 270 million inhabitants. In recent decades, Indonesia has made significant progress in health outcomes, but several challenges remain, particularly in maternal health, nutrition, communicable diseases such as tuberculosis, and an increasing health burden from non-communicable diseases [16]. Indonesia spends relatively little on health (3% of GDP, 2020), compared to the average of upper middle-income countries (7% of GDP, 2020) [7]. Approximately 57% of all health expenditure is from the government, with one-third from household OOP payments [6, 17].

### Data collection

This research was part of the “Equity and Health Care Financing in Indonesia” (ENHANCE) Study [18]. The over-arching goal of ENHANCE is to assess the progress being made by Indonesia towards UHC and the impact of major reforms such as the JKN on financial protection. A detailed description of the study aims and methods can be found in the published protocol [18]. The ENHANCE study administrated two-round household survey in 10 of the 34 provinces in Indonesia. These 10 provinces accounted for about 74% of the Indonesian population and allowed a comprehensive assessment of the impact of the JKN on households’ health spending. The first wave of data collection occurred in February – April 2018, and the second wave was carried out in August – October 2019. The selection of provinces was done purposively to capture Indonesia’s different socioeconomic conditions and demographic characteristics. The key considerations for selecting provinces were population size and location. The selection of districts within provinces was based on the geographic location (rural/urban) and fiscal capacity index (FCI) as defined by the Indonesian Ministry of Finance, [19].

$$\begin{array}{l}Fiscal\,capacity\,index\,of\,province\,\,i\,\\= \,\frac{{Total\,revenu{e_i} - (Mandatory\,spendin{g_i}\, + \,Specific\,allocation\,spendin{g_i})}}{{Total\,provincial\,fiscal\,capacity/Number\,of\,provinces}}\end{array}$$

This index is published every year and compares regional governments in terms of their capacity to finance a minimum standard of services. The index is used for the distribution of the national budget to regional (both Provincial and District) governments to promote equity (i.e. providing a greater central budget allocation to provinces with lower fiscal capacity ) [19]. In the study provinces, households were selected using a systematic random sampling procedure without any exclusion criteria. First, three districts were selected from each of the ten sample provinces and then classified as having either high, moderate, or low fiscal capacity based on the Regional Fiscal Capacity Map [19]. In each selected district, two sub-districts and four villages (two villages per sub-district) were randomly sampled using a list of sub-districts and villages as the sampling frame. Finally, in each village, we selected neighbourhoods from which households were proportionally selected. Overall, 7,554 households were surveyed in the first wave (2018), but not all could be traced in the second wave, leading to 6,445 households (85% of the first wave) being re-interviewed in 2019. Full details of the sampling procedure and data collection techniques are published elsewhere [18]. In this study, we analysed data from the 6,445 households that completed both rounds of survey (85% of the total number of households interviewed in the first wave).

### Data variables

We collected information on socioeconomic status, health insurance coverage, health care utilization and OOP health expenditures for each health service used. There were two types of health insurance coverage: public (JKN) and private health insurance. The health care utilization data included outpatient and inpatient care. Outpatient care represented the utilization of outpatient services in the last month from primary and secondary providers for each member of the household and all associated OOP expenditure. Inpatient care represented the utilization of inpatient services over the last year and all related OOP expenditure. OOP expenditure included fees for medical consultation, laboratory tests, radiology, drugs, medical supplies, transportation, food, and unofficial payments (e.g. tips to health workers). Unofficial payments can be initiated either by patients who believe that they will receive more attention and better treatment or by employees of public health care services [20]. In the 2019 survey, a question on hospital room charges was added to the calculation of OOP payments for inpatient care. In the survey, we also collected data on household consumption expenditure in the past month such as spending on education, utilities and transportation.

### Analysis

The WHO and the World Bank define CHS as household expenditure on health care greater than 10% or greater than 25% of total household consumption expenditure. In the baseline analysis, the incidence of CHS was calculated as the proportion of households with OOP spending exceeding 10% of total household consumption expenditure, which is applied by SDG indicator 3.8.2 [1]. In the sensitivity analysis, we assessed the incidence of CHS based on the higher threshold of 25% of total household expenditure. We first aggregated individual health care utilization at the household level by adding up the number of outpatient visits and inpatient days for each household member. Similarly, we summed the OOP payments of each member to derive the total OOP payments within the household. OOP payments for outpatient care in the last month were multiplied by twelve to derive annual OOP payments. Total household consumption expenditure included eight common items: food and non-food expenditure (schooling, electricity, water, transportation, fuel, health care, social events). Annual household consumption expenditure was calculated by multiplying monthly expenditure by twelve. Sensitivity analysis excluding data on hospital room charges from OOP payments collected only in 2019 was conducted. Chi-squared tests were used to compare the incidences of CHS across subgroups for each household characteristic.

We then used logistic regression models to assess factors associated with incurring CHS in each wave. The dependent variable was whether the household experienced CHS (yes or no). The independent variables included: residence type (urban vs. rural as reference), occupation of household head (civil servant, private employee, self-employed, vs. unemployed as reference), household expenditure quintile (the poorest as the reference vs. poorer, middle, richer, and richest), health insurance coverage (JKN, private health insurance, vs. no health insurance coverage as reference), number of children under five years of age (one or more vs. none as reference), number of adults aged 60 + years (one or more vs. none as reference), and type of health care providers for outpatient or inpatient care (private, not using care, vs. public as reference). We also conducted a pooled data analysis using data from all households in both survey rounds of survey (7,554 households in the first wave, plus 6,445 households in the second wave). Again, a logistic regression model was used to predict the likelihood of incurring CHS and study round was included as an independent variable to assess the change over time in CHS. An interaction term between survey round and health insurance coverage was included. Variability between villages was first examined but not included in the final model as the variability was not statistically significant. All analyses were performed using STATA (version 15).

## Results

### Baseline analysis

The descriptive statistics of the matched 6,445 households in 2018 and in 2019 are shown in Table 1. The proportion of households with JKN membership increased from 64% to 2018 to 73% in 2019 and was highest among households where the head was a civil servant (88% in 2018, 95% in 2019, Appendix Table A1). The average household expenditure was higher in all five wealth quintiles in 2019 compared to 2018. Twenty-eight percent of households had one or more children under five years of age in 2018, dropping to 25% in 2019. The proportion of households with one or more adults aged over 60 years of age increased from 31% to 2018 to 34% in 2019. More than half of all households sought outpatient care in private health facilities in both 2018 and 2019. However, the high use of public hospitals for inpatient care in 2018 changed in 2019 with utilization evenly split between public and private hospitals. Among the 6,445 households that completed both rounds of the survey, 24 individuals in the first wave and 20 individuals in the second wave reported not seeking care when they were sick because they could not afford to pay medical fees.

**Table 1:** Descriptive statistics of the distribution of households in the ENHANCE Panel Surveys of 2018 and 2019 (N = 6,445)

Variables	2018 n (%)	2019 n (%)
Health insurance coverage		
Public (JKN)	4,126 (64.0)	4,672 (72.5)
Private	109 (1.7)	72 (1.1)
No coverage	2,210 (34.3)	1,701 (26.4)
Residence		
Urban	3,951 (61.3)	3,668 (56.9)
Rural	2,494 (38.7)	2,777 (43.1)
Employment of the head of household		
Unemployed	474 (7.6)	551 (8.7)
Civil servant	349 (5.6)	350 (5.5)
Private employee	731 (11.7)	863 (13.6)
Self-employed (informal sector)	4,712 (75.2)	4,570 (72.2)
Average household monthly expenditures (in IDR, 000)		
Poorest, Q1	871	909
Poorer, Q2	1,537	1,698
Middle, Q3	2,048	2,364
Richer, Q4	2,688	3,353
Richest, Q5	5,303	6,664
Number of children under five years old		
None	4,626 (71.8)	4,829 (74.9)
One or more	1,819 (28.2)	1,616 (25.1)
Number of adult aged 60 +		
None	4,425 (68.7)	4,256 (66.0)
One or more	2,020 (31.3)	2,189 (34.0)
Distribution of outpatient care in the last month		
Public only	1,147 (17.8)	952 (14.8)
Private	1,777 (25.6)	1,327 (20.6)
Not using outpatient care	3,521 (54.6)	4,166 (64.6)
Distribution of inpatient care in the last year		
Public only	694 (10.8)	510 (7.9)
Private	455 (7.1)	465 (7.2)
Not using inpatient care	5,296 (82.2)	5,470 (84.9)
Number of individuals and reasons for not seeking care	108	43
The pain is not severe	48	5
Unable to pay medical expenses	24	20
Long and long queues	22	4
Other	14	14

JKN, Jaminan Kesehatan Nasional; IDR, Indonesian Rupiah

Table 2 shows the OOP payments for outpatient and inpatient care by household characteristics. The average OOP payment by households with JKN coverage decreased from IDR 268,000 in 2018 to IDR 128,000 in 2019 for outpatient care. OOP payments for inpatient care decreased from IDR 1,596,000 in 2018 to IDR 1,347,000 in 2019. Private health insurance holders, by contrast, experienced an increase in OOP payments for both outpatient and inpatient care in 2019 compared to 2018. Uninsured households incurred relatively small OOP payments for outpatient care while experiencing an increase in inpatient OOP payments. Households with higher consumption expenditure incurred much higher OOP payments compared to lower expenditure groups. As expected, households utilising private health care providers, on average, incurred more OOP costs compared to those using public health care providers. For outpatient care, most OOP costs were incurred on drugs and diagnostics (laboratory and radiology examinations). The bulk of OOP spending for inpatient care was on drugs and diagnostics.

**Table 2:** Average out-of-pocket payments for outpatient and inpatient care in 2018 and 2019 (in IDR 000)*

	Outpatient				Inpatient			
	2018		2019		2018		2019	
	Mean	SD	Mean	SD	Mean	SD	Mean	SD
Health insurance coverage								
Public (JKN)	268	3,244	128	545	1,596	5,041	1,347	5,159
Private	294	421	1,007	2,515	1,863	4,548	7,849	22,200
No coverage	215	831	138	310	2,328	3,494	2,970	4,315
Household monthly expenditure quintile								
Poorest, Q1	118	576	92	341	1,481	3,257	1,046	2,600
Poorer, Q2	84	129	135	639	1,124	2,103	1,125	2,211
Middle, Q3	106	193	127	446	1,346	4,052	1,225	2,652
Richer, Q4	370	453	131	655	1,843	5,287	1,261	3,219
Richest, Q5	485	3,026	186	541	2,668	6,190	2,731	9,380
Type of health provider								
Public only	124	619	78	443	1,339	3,606	1,267	6,132
Private	301	3,032	169	594	2,410	5,846	1,981	4,854
OOP components								
Doctors’ fees	187	477	74	108	3,195	3,922	818	1,374
Administration	138	617	82	476	2,346	3,307	2,143	5,212
Drugs and diagnostics	289	3,047	165	591	3,018	6,730	2,127	4,963
Room^	NA	NA	NA	NA	NA	NA	1,830	2038
Transportation	45	381	22	36	178	557	295	4,594
Food	100	549	72	500	365	450	316	833
Informal payments	5	7	7	37	66	272	57	398

JKN, Jaminan Kesehatan Nasional; OOP, out-of-pocket; SD, standard deviation

*This analysis was conducted for households that reported OOP payments

^OOP for room charges was only asked for inpatient services in 2019

Table 3 presents the incidence of CHS at the 10% expenditure threshold in 2018 and 2019. The overall incidence of CHS is shown to have dropped from 7.9% to 2018 to 4.4% in 2019. The incidence of CHS among JKN members dropped from 7.0% to 2018 to 4.0% in 2019 and was significantly lower compared to households with private health insurance and households without insurance coverage. Households in rural areas experienced a significantly higher incidence of CHS than those in urban areas in 2018 (9.7% vs. 6.8%, P < 0.001), while the gap narrowed in 2019 (4.9% vs. 4.1%). Households where the household head was unemployed incurred an incidence of CHS at 13% in 2018, which was significantly higher than other households. In 2019, the difference was not significantly different among households with different employment statuses. The incidence of CHS was significantly higher among lowest expenditure quintiles in both years (9.7% and 5.6%). Households with one or more children under five had a significantly higher incidence of CHS compared to households with no children in both waves. The incidence of CHS was also significantly higher among households with elderly members in both years. As expected, using private health care providers was associated with a much higher incidence of CHS than seeking care from public health care providers, for both outpatient and inpatient services in both waves.

**Table 3:** Incidence of catastrophic health spending (%) at 10% expenditure threshold (N = 6,445)

	2018 % (95% CI)		2019 % (95% CI)	
**All households**	7.9 (7.3, 8.6)		4.4 (4.0, 5.0)	
**Health insurance coverage**		P = 0.001		P = 0.031
Public (JKN)	7.0 (6.2, 7.8)		4.0 (3.5, 4.6)	
Private	10.1 (5.1, 17.3)		5.6 (1.5, 13.6)	
No coverage	9.6 (8.4, 10.9)		5.5 (4.5, 6.7)	
**Residence**		P < 0.001		P = 0.095
Urban	6.8 (6.1, 7.7)		4.1 (3.5, 4.8)	
Rural	9.7 (8.5, 10.9)		4.9 (4.2, 5.8)	
**Employment of the head of household**		P < 0.001		P = 0.135
Unemployed	13.0 (10.1, 16.4)		6.4 (4.5, 8.8)	
Civil servant	11.2 (8.1, 15.0)		4.9 (2.9, 7.9)	
Private employee	5.9 (4.3, 7.8)		4.1 (2.8, 5.6)	
Self-employed	7.6 (6.8, 8.4)		4.3 (3.7, 4.9)	
**Household monthly expenditure quintile**		P = 0.002		P = 0.012
Poorest, Q1	9.7 (8.2, 11.5)		5.6 (4.4, 7.0)	
Poorer, Q2	7.2 (5.9, 8.7)		4.7 (3.6, 6.0)	
Middle, Q3	6.4 (5.1, 7.9)		4.4 (3.4, 5.7)	
Richer, Q4	6.9 (5.5, 8.4)		3.9 (2.9, 5.1)	
Richest, Q5	9.5 (7.9, 11.2)		3.6 (2.6, 4.7)	
**Number of children under five years old**		P = 0.002		P < 0.001
None	7.3 (6.5, 8.1)		3.7 (3.2, 4.3)	
One or more	9.6 (8.3, 11.1)		6.6 (5.4, 7.9)	
**Number of adult aged 60+**		P < 0.001		P = 0.040
None	6.9 (6.2, 7.8)		4.1 (3.5, 4.7)	
One or more	10.1 (8.8, 11.5)		5.2 (4.3, 6.2)	
**Type of outpatient care provider**		P < 0.001		P < 0.001
Public only	7.6 (6.1, 9.3)		4.5 (3.3, 6.0)	
Private	19.8 (18.0, 21.7)		14.3 (12.5, 16.3)	
Not using outpatient care	2.0 (1.6, 2.6)		1.3 (1.0, 1.7)	
**Type of inpatient care provider**		P = 0.001		P < 0.001
Public only	19.3 (16.4, 22.4)		10.2 (7.7, 13.2)	
Private	27.7 (23.6, 32.1)		18.1 (14.7, 22.0)	
Not using inpatient care	4.7 (4.2, 5.4)		2.7 (2.3, 3.2)	

JKN, Jaminan Kesehatan Nasional

Chi-squared tests were conducted for comparing incidences of CHS across subgroups.

Factors associated with incurring CHS at the 10% threshold are summarised in Table 4. JKN membership was associated with a significantly lower likelihood of incurring CHS in both 2018 and 2019 compared to households without any health insurance. Households in urban areas were less likely to incur CHS than those living in rural areas in 2018. The odds of incurring CHS were significantly lower among households where the head was either a private employee or self-employed compared to households where the head was unemployed in 2018. In both years, households with lowest consumption expenditure demonstrated a significantly higher odds of experiencing CHS compared with richer households. The number of children and elderly members within households did not significantly impact the probability of incurring CHS in both years. On the other hand, households seeking health care from private providers were more likely to experience CHS in both 2018 and 2019.

**Table 4:** Determinants of catastrophic health spending at 10% threshold, 2018–2019 (N = 6,445)

Variables			2018 Odds ratio (95% CI)	P-value	2019 Odds ratio (95% CI)	P-value
Health insurance coverage						
No coverage			ref		ref	
Public (JKN)			0.61 (0.49–0.77)	< 0.001	0.48 (0.35–0.65)	< 0.001
Private			0.86 (0.39–1.86)	0.694	0.82 (0.25–2.69)	0.748
Location						
Rural			ref		ref	
Urban			0.66 (0.54–0.82)	< 0.001	1.13 (0.86–1.48)	0.368
Employment of the head of household						
Unemployment			ref		ref	
Civil servant			1.20 (0.73–1.98)	0.476	1.09 (0.55–2.14)	0.804
Private employee			0.52 (0.32–0.83)	0.006	0.83 (0.48–1.44)	0.507
Self-employed			0.71 (0.50–0.99)	0.047	0.81 (0.53–1.24)	0.334
Household income quintile						
Poorest (Q1)			ref		ref	
Poorer (Q2)			0.59 (0.43–0.81)	0.001	0.50 (0.33–0.74)	0.001
Middle (Q3)			0.43 (0.31–0.59)	< 0.001	0.47 (0.31–0.70)	< 0.001
Richer (Q4)			0.47 (0.34–0.66)	< 0.001	0.31 (0.20–0.47)	< 0.001
Richest (Q5)			0.53 (0.39–0.73)	< 0.001	0.23 (0.15–0.37)	< 0.001
Number of children under five years old						
None			ref		ref	
One or more			0.99 (0.79–1.24)	0.923	1.21 (0.91–1.61)	0.187
Number of residents aged 60+						
None			ref		ref	
One or more			1.18 (0.95–1.47)	0.138	1.10 (0.83–1.47)	0.499
Outpatient care provider						
Public			ref		ref	
Private			3.70 (2.80–4.90)	< 0.001	3.96 (2.73–5.74)	< 0.001
Not using outpatient care			0.29 (0.21–0.40)	< 0.001	0.27 (0.18–0.41)	< 0.001
Inpatient care provider (ref = public)						
Public			ref		ref	
Private			1.34 (0.97–1.86)	0.079	1.70 (1.11–2.59)	0.014
Not using inpatient care			0.16 (0.12–0.21)	< 0.001	0.17 (0.12–0.25)	< 0.001

JKN, Jaminan Kesehatan Nasional; CI, confidence interval

### Pooled data analysis

The pooled data analyses with and without an interaction term between insurance type and survey rounds are summarized in Table 5. The interaction term was not statistically significant. JKN membership, residing in an urban area, being a private employee and self-employment became protective factors for incurring CHS. Households with lowest household expenditure had a higher likelihood of incurring CHS compared with richer households. Households visiting private facilities were more likely to experience CHS than households visiting public facilities only. The overall trend of CHS occurrence fell significantly over the two time points (OR = 0.66, P < 0.001).

**Table 5:** Pooled data analysis predicting the probability of incurring catastrophic health spending at 10% threshold (N = 13,997)*

Variables	Odds ratio (95% CI)	P-value	Odds ratio (95% CI)	P-value
Health insurance coverage				
No coverage	ref			
Public (JKN)	0.52 (0.42–0.65)	< 0.001	0.55 (0.42–0.72)^a^	< 0.001
Private	0.91 (0.43–1.94)	0.814	0.89 (0.37–2.17)^b^	0.806
Location				
Rural	ref		ref	
Urban	0.79 (0.64–0.96)	0.019	0.79 (0.64–0.96)	0.020
Employment of the head of household				
Unemployment	ref		ref	
Civil servant	1.26 (0.78–2.06)	0.345	1.26 (0.78–2.06)	0.346
Private employee	0.59 (0.38–0.90)	0.016	0.59 (0.38–0.90)	0.015
Self-employed	0.72 (0.52–0.99)	0.045	0.72 (0.52–0.99)	0.045
Household income quintile				
Poorest (Q1)	ref		ref	
Poorer (Q2)	0.51 (0.38–0.69)	< 0.001	0.51 (0.38–0.68)	< 0.001
Middle (Q3)	0.38 (0.28–0.51)	< 0.001	0.38 (0.28–0.52)	< 0.001
Richer (Q4)	0.35 (0.26–0.48)	< 0.001	0.35 (0.26–0.48)	< 0.001
Richest (Q5)	0.34 (0.25–0.47)	< 0.001	0.34 (0.25–0.47)	< 0.001
Number of children under five years old				
None	ref		ref	
One or more children	1.09 (0.88–1.34)	0.440	1.09 (0.88–1.35)	0.431
Number of residents aged 60+				
None	ref		ref	
One or more	1.21 (0.98–1.49)	0.079	1.21 (0.98–1.49)	0.079
Outpatient care provider				
Public				
Private	4.80 (3.63–6.35)	< 0.001	4.80 (3.63–6.34)	< 0.001
Not using outpatient care	0.22 (0.16–0.30)	< 0.001	0.22 (0.16–0.30)	< 0.001
Inpatient care provider				
Public	ref		ref	
Private	1.68 (1.22–2.30)	0.001	1.68 (1.23–2.30)	0.001
Not using inpatient care	0.11 (0.08–0.15)	< 0.001	0.11 (0.08–0.15)	< 0.001
Survey round				
First wave	ref			
Second wave	0.66 (0.55–0.80)	< 0.001	0.73 (0.53–1.01)^a^	0.061
Insurance type # survey round				
No coverage # first wave			ref	
JKN # second wave			0.85 (0.57–1.28)^b^	0.446
Private # second wave			1.09 (0.22–5.43)^c^	0.918
JKN, Jaminan Kesehatan Nasional; CI, confidence interval; ref, reference group *Two observations from first wave were excluded due to missing data. ^a^ comparison is made between households with no health insurance in the second wave and households with no health insurance in the first wave (reference) ^b^ comparison is made between households with JKN in the second wave and households with no health insurance in the first wave (reference) ^c^ comparison is made between households with private health insurance in the second wave and households with no health insurance in the first wave (reference)				

### Sensitivity analysis

When hospital room charges were excluded from the calculation of OOP payments in 2019, the incidence of CHS in 2019 remained similar to the baseline analysis (Appendix Table A2). When the incidence of CHS was defined as the proportion of households spending over 25% of their household consumption expenditure on OOP health care payments, the incidence of CHS fell from 2.7% to 2018 to 1.5% in 2019 (Appendix Table A3). Self-employment was associated with significantly lower likelihood of incurring CHS at the 25% threshold in both years (Appendix Table A4). Being insured under the JKN, living in an urban area, and being a private employee were protective factors in 2018, but not in 2019. On the other hand, the richer households (Q4 and Q5) had significantly lower odds of incurring CHS than the poorest households (Q1) at the 25% threshold in both years. Households with children under five and households with elderly members were more likely to experience CHS in 2019. Results from the pooled data analysis showed that at the 25% threshold, JKN membership, living in an urban area, being a private employee/self-employed, living in richer households, and not using health care, were associated with a significantly lower likelihood of experiencing CHS (Appendix Table A5). Overall, households in the second wave had a significantly lower odds of incurring CHS at the 25% threshold compared to households in the first wave (OR = 0.67, P = 0.005).

## Discussion

This study investigated the incidence, determinants and trend of CHS under the JKN in Indonesia. A major finding of this study was that the overall incidence of CHS fell from 7.9% to 2018 to 4.4% in 2019. Among households covered by the JKN, the incidence of CHS was 7.0% in 2018 and 4.0% in 2019, which was lower than estimates among households without insurance and households covered by private insurance. Overall, the incidence of CHS displayed a declining trend over the study period and the change was statistically significant.

Our estimate of the incidence of CHS in 2018 is higher than the number published by the WHO and World Bank in 2021 [5]. Using the 2017 National Socioeconomic Survey (Survei Sosial Ekonomi Nasional, SUSENAS), the WHO and World Bank reported that the incidence of CHS at the 10% threshold was 4.5% in 2017 in Indonesia. But it should be noted that the SUSENAS survey did not collect OOP payments on diagnostic procedures, transportation, food and informal payments (Appendix Table A6). Moreover, the recall period was defined as the past month for all OOP expenditure in the SUSENAS, which might have underestimated the total OOP payments, especially the inpatient OOP costs.

Our study found that membership of the JKN was associated with a lower incidence of CHS during the study period. This finding is consistent with previous Indonesian studies evaluating financial protection under the JKN Program [21–23]. However, the level of protection in Indonesia was shown to be inadequate to eliminate OOP payments and in turn CHS for some JKN members. In theory, JKN members should not incur OOP payments when they visit health facilities within the JKN network. But there is evidence that the cost of medical procedures is often higher than the capitation payment and INA-CBG tariffs [24, 25]. As a result, households may still face the burden of OOP health payments even if they are insured under the JKN. Moreover, a new government health financing regulation (Presidential Regulation No. 82) implemented in 2018 enables JKN members to pay OOP for up to 75% of INA-CBG tariffs for an upgrade to an executive clinic or VIP room [12]. This may also explain why some households incurred OOP payments and CHS despite being members of the JKN. However, due to the endogeneity of the JKN program - that arises from the choice to be insured, eligibility for insurance, and differences in the health status of individuals - the causal effect of the JKN on CHS could not be determined using the data collected by this study.

Our study found that the poorest households experienced the highest incidence of CHS and had higher odds of experiencing CHS. This finding is consistent with findings reported in several other countries, including Kenya [26, 27] and China [28]. Despite lower OOP payments among the poorest households, these households still incurred a higher incidence of CHS due to relatively low income. Our data also showed that OOP expenditure was the highest among the richest households. It is likely that richer households are more willing to pay upgrade fees under the JKN scheme. In Indonesia, more than half of the roughly 3,000 hospitals are private hospitals [29] and it is likely that richer households make more use of private health facilities which are more expensive. Richer households may also be willing to pay more for care that is perceived to be of better quality. Improved routine measurement of the quality of care is critical to understanding whether the rich may be spending more to access better quality of care in Indonesia and in other low- and middle-income countries [30–32].

The pooled data analysis showed that households in urban areas had lower odds of incurring CHS. This finding is consistent with studies in Peru [33], Bangladesh [34] and Senegal [35]. It also aligns with other studies from Indonesia reporting lower household expenditure (usually used as a proxy of income) in rural areas [36]. Moreover, data published by the Statistics Indonesia (Badan Pusat Statistik - BPS) revealed that between 2014 and 2021, the average monthly household expenditure of urban households increased from IDR 979,000 in 2014 to IDR 1,487,000 in 2021, or by 70%. However, the average monthly household expenditure in rural areas increased by only 52%, from IDR 573,000 in 2014 to IDR 971,000 in 2021 [37]. With the same level of health care costs, the incidence of CHS would be lower for households in urban areas due to the higher average income. In the pooled data analysis, households with a head employed in the private sector or self-employed were less likely to incur CHS compared to households with an unemployed head. Other studies have reported similar findings that having a household head who was unemployed significantly increased the odds of incurring CHS [27, 33]. Households with an employed head will most likely have a higher income than those whose head is unemployed and thus with the same level of OOP spending, these households would have lower levels of CHS.

In the 6,445 households that participated in both surveys, altogether 44 respondents reported not seeking care when they were sick with the main reason being that care was unaffordable. Though forgone health care (or unmet health care needs) due to financial barriers did not appear to be a major issue in the surveyed households, this may be due to the one month recall period used in this study compared to studies in OECD countries which typically ask about spending over the last 12 months [38]. For future studies, we recommend that household surveys include unmet health care needs and also collect information on the reasons for not seeking care.

A number of policy recommendations can be made based on the findings of this study. Firstly, to further reduce OOP spending, we recommend that the government reviews its capitation payments and INA-CBG tariffs to ensure these match the real costs of delivering health care services. Strategic purchasing of health services from providers based on capitation payments and INA-CBG tariffs may also help reduce OOP spending, as health providers who charge patients beyond the maximum cost-sharing threshold face the risk of non-renewal of their contract with government. Secondly, claims submitted to the JKN Administrator by health care providers currently do not include OOP payments for services that are not covered under the JKN or any top-up payments for service upgrades. Inclusion of this information can provide a much better understanding of the financial impact of health spending by households. Thus, we recommend that the JKN Administrator collaborates with BPS to include questions on these types of OOP payments in its annual SUSENAS survey. Collecting this information will also facilitate international comparison with other UN member states for monitoring progress towards the SDGs (specifically the indicators for SDG3.8.2). Finally, a more in-depth analysis is needed to identify those areas of health spending that are placing greatest financial burden on households. For example, it is likely that OOP payments for drugs are more burdensome compared to OOP payments for medical fees, since the prices of drugs tend to increase in line with the exchange rate. In this situation the government could consider implementing a safety net scheme for low-income members of the JKN.

Our study has some limitations. The study coincided with the issuance of a new government health financing regulation allowing JKN members to pay up to 75% of the CBG tariff to upgrade inpatient care to a higher class of hospitalization or change to a “VIP room” or executive clinic. This regulation is believed to have contributed to the high OOP spending by high income groups including some JKN members. In this case, the incidence of CHS may not be the most appropriate measure, as it does not take into account voluntary OOP payments towards such health services. Secondly, this study cannot capture the potential effects of seasonality on service utilization rate and household health spending, as the two survey rounds were conducted in different months of the year. Therefore, cautious interpretation is needed on the changes of CHS incidence between 2018 and 2019. Moreover, outpatient OOP spending during the survey month was multiplied by 12 in order to derive an estimate of annual spending on outpatient services. Again, seasonal variations in monthly OOP payments for outpatient visits were unable to be captured under this approach. Total household consumption expenditure may have also been underestimated as our survey covered only eight common items and excluded other potentially relevant items such as clothing, rent and home appliances. Therefore, interpretation of CHS incidence needs to take into account these complexities. Although the survey covered 74% of the Indonesian population, the findings of our study may not be nationally representative, as we use purposive sampling in order to capture different socioeconomic conditions and demographic characteristics. Finally, this study may be limited by sample selectivity due to attrition. In the analyses, we only included households that could be followed up in the second wave. The proportion of households with non-PBI members and private insurance was significantly higher among those lost to follow-up. Households lost to follow-up were also wealthier. Thus, our estimates of incidence of CHS may be biased due to attrition. Despite these limitations, this study has provided insights into the incidence of CHS and its determinants for the same households at two time points, which has never been done before in the context of Indonesia.

## Conclusions

In this study, we aimed to assess the incidence of CHS, factors associated with incurring CHS and trends in CHS under the JKN in Indonesia. This study demonstrated that the overall incidence of CHS decreased in Indonesia between 2018 and 2019. However, a higher incidence of CHS was observed among households in rural areas, non-JKN members, households where household heads were unemployed, and the poorest households. The inequality among those experiencing a high incidence of CHS could be potentially addressed by ensuring health policies such as providing a safety net covering health care expense to low-income households and expanding JKN coverage. While households with JKN membership were found to have a lower incidence of CHS compared to households with private insurance and households without health insurance, the national health insurance scheme has not provided adequate protection to all its members. OOP payments for health care and the risk of CHS still loom high among JKN members. While the higher OOP payments among wealthier households may not appear to be of major concern from an equity perspective, it does require further investigation to assess if these OOP payments are caused by unnecessary prescription of tests and treatment through supplier induced demand. We also need further research to investigate whether CHS pushes households below the poverty line.

### Electronic supplementary material

Below is the link to the electronic supplementary material.

Supplementary Material 1

## Data Availability

Post-processing source data and supplementary data are presented within this study. Proposal to access to the ENHANCE survey datasets should be directed to the corresponding author to gain access. Data requestors will need to sign a data access agreement.

## List of abbreviations

UHC: Universal health coverage
SDGs: Sustainable development goals
UN: United Nations
OOP: Out-of-pocket
NHA: National Health Accounts
CHS: Catastrophic health spending
WHO: World Health Organization
JKN: Jaminan Kesehatan Nasional
BPJS: Badan Penyelenggara Jaminan Sosial
INA-CBG: Indonesian Case Base Groups
IDR: Indonesian Rupiah
SUSENAS: Survei Sosial Ekonomi Nasional
