# Supplementary material for: Incidence of catastrophic health spending in Indonesia: insights from a Household Panel Study 2018–2019
Source: Int J Equity Health. 2023 Sep 6;22:185. doi: 10.1186/s12939-023-01980-w (PMC10483778; doi:10.1186/s12939-023-01980-w)
Supplement: Supplementary file 1 — Supplementary Material 1 [file 12939_2023_1980_MOESM1_ESM.docx]

| Variables | 2018  n (%) | 2019  n (%) |
| --- | --- | --- |
| Overall | 4,126 (64.0) | 4,672 (72.5) |
| Residence |  |  |
| Urban | 2,754 (69.7) | 2,895 (78.9) |
| Rural | 1,372 (55.0) | 1,777 (64.0) |
| Employment of the head of household |  |  |
| Unemployed | 326 (68.8) | 422 (76.6) |
| Civil servant | 307 (88.0) | 331 (94.6) |
| Private employee | 547 (74.8) | 687 (79.6) |
| Self-employed (informal sector) | 2,836 (60.2) | 3,232 (70.7) |
| Average household monthly expenditures |  |  |
| Poorest, Q1 | 832 (64.6) | 913 (70.7) |
| Poorer, Q2 | 828 (63.5) | 882 (68.4) |
| Middle, Q3 | 783 (61.2) | 918 (71.3) |
| Richer, Q4 | 835 (65.1) | 975 (75.6) |
| Richest, Q5 | 848 (65.8) | 984 (76.3) |
| Number of children under 5 years in the household |  |  |
| None | 3,069 (66.3) | 3,525 (73.0) |
| 1 or more | 1,057 (58.1) | 1,147 (71.0) |
| Number of adult aged 60+ in the household |  |  |
| None | 2,752 (62.2) | 3,208 (71.2) |
| 1 or more | 1,374 (68.0) | 1,644 (75.1) |

**Table A1. JKN coverage by household characteristics (N=6,445)**

**Table A2. Incidence of catastrophic health spending (%) at 10% expenditure threshold in Wave 2 (N=6,445)**

|  | 2019 | | 2019* | |
| --- | --- | --- | --- | --- |
| All households | 4.4 |  | 4.2 |  |
| Health insurance coverage |  | p=0.031 |  | p=0.118 |
| Public (JKN) | 4 |  | 3.8 |  |
| Private | 5.6 |  | 5.6 |  |
| No coverage | 5.5 |  | 5 |  |
| Residence |  | p=0.095 |  | p=0.080 |
| Urban | 4.1 |  | 3.8 |  |
| Rural | 4.9 |  | 4.7 |  |
| Employment of the head of household |  | p=0.135 |  | p=0.150 |
| Unemployed | 6.4 |  | 6 |  |
| Civil servant | 4.9 |  | 4.3 |  |
| Private employee | 4.1 |  | 3.8 |  |
| Self-employed | 4.3 |  | 4 |  |
| Household monthly expenditure quintile |  | P=0.012 |  | P=0.020 |
| Poorest, Q1 | 5.6 |  | 5.6 |  |
| Poorer, Q2 | 4.7 |  | 4.4 |  |
| Middle, Q3 | 4.4 |  | 4 |  |
| Richer, Q4 | 3.9 |  | 3.7 |  |
| Richest, Q5 | 3.6 |  | 3.1 |  |
| Household with children under 5 years old |  | P<0.001 |  | P<0.001 |
| None | 3.7 |  | 3.5 |  |
| 1 or more | 6.6 |  | 6.1 |  |
| Household with adult aged 60+ |  | P=0.040 |  | P=0.021 |
| None | 4.1 |  | 3.7 |  |
| 1 or more | 5.2 |  | 5 |  |
| Type of outpatient care provider |  | P<0.001 |  | P<0.001 |
| Public | 4.5 |  | 4.4 |  |
| Private | 14.3 |  | 14.1 |  |
| Type of inpatient care provider |  | P<0.001 |  | P P<0.001 |
| Public | 10.2 |  | 9.4 |  |
| Private | 18.1 |  | 14.9 |  |
| JKN, Jaminan Kesehatan Nasional  *Excluded room rent fees from the calculation of out-of-pocket payment | | | | |

**Table A3. Incidence of CHS (%) at 25% expenditure thresholds (N=6,445)**

|  | **2018** |  | **2019*** |  |
| --- | --- | --- | --- | --- |
| All households | 2.7 |  | 1.5 |  |
| Health insurance coverage |  | P=0.039 |  | P=0.128 |
| Public (JKN) | 2.4 |  | 1.4 |  |
| Private | 1.8 |  | 4.2 |  |
| No coverage | 3.4 |  | 1.6 |  |
| Residence |  | P=0.001 |  | P=0.005 |
| Urban | 2.2 |  | 1.1 |  |
| Rural | 3.6 |  | 2.0 |  |
| Employment of the head of household |  | P=0.001 |  | P=0.003 |
| Unemployed | 6.0 |  | 3.3 |  |
| Civil servant | 4.9 |  | 1.7 |  |
| Private employee | 2.2 |  | 1.5 |  |
| Self-employed | 2.4 |  | 1.3 |  |
| Household income quintile |  | P<0.001 |  | P=0.109 |
| Poorest, Q1 | 3.9 |  | 1.6 |  |
| Poorer, Q2 | 1.8 |  | 2.2 |  |
| Middle, Q3 | 1.9 |  | 1.5 |  |
| Richer, Q4 | 2.3 |  | 1.0 |  |
| Richest, Q5 | 3.9 |  | 1.2 |  |
| Household with children under 5 years old |  | P=0.770 |  | P=0.001 |
| None | 2.8 |  | 1.2 |  |
| 1 or more | 2.6 |  | 2.5 |  |
| Household with adult aged 60+ |  | P=0.103 |  | P=0.001 |
| None | 2.5 |  | 1.1 |  |
| 1 or more | 3.2 |  | 2.2 |  |
| Type of health provider (outpatient) |  | P<0.001 |  | P<0.001 |
| Not seeking care | 0.8 |  | 0.3 |  |
| Public | 2.4 |  | 1.9 |  |
| Private | 6.7 |  | 5.0 |  |
| Type of health provider (inpatient) |  | P<0.001 |  | P<0.001 |
| Not seeking care | 1.5 |  | 0.9 |  |
| Public | 7.6 |  | 2.6 |  |
| Private | 10.6 |  | 6.9 |  |
| JKN, Jaminan Kesehatan Nasional  *Excluded room rent fees from the calculation of out-of-pocket payment | | | | |

**Table A4. Determinants of CHS at 25% threshold, 2018 – 2019 (N=6,445)**

| **Variables** | | | **2018**  **Odds ratio (95% CI)** | **P-value** | **2019**  **Odds ratio (95% CI)** | **P-value** |
| --- | --- | --- | --- | --- | --- | --- |
| Health insurance coverage | | |  |  |  |  |
| no coverage | | | ref |  | ref |  |
| Public (JKN) | | | 0.58 (0.41 – 0.81) | 0.002 | 0.71 (0.43-1.19) | 0.195 |
| Private | | | 0.28 (0.06 – 1.28) | 0.102 | 2.66 (0.68-10.45) | 0.162 |
| Location | | |  |  |  |  |
| Rural | | | ref |  | ref |  |
| Urban | | | 0.58 (0.42-0.81) | 0.001 | 0.71 (0.46-1.10) | 0.126 |
| Employment of the head of household | | |  |  |  |  |
| Unemployed | | | ref |  | ref |  |
| Civil servant | | | 1.09 (0.55-2.17) | 0.810 | 0.74 (0.27-2.04) | 0.565 |
| Private employee | | | 0.50 (0.25-1.00) | 0.048 | 0.65 (0.29-1.47) | 0.302 |
| Self-employed | | | 0.49 (0.30-0.79) | 0.003 | 0.50 (0.27-0.90) | 0.022 |
| Household income quintile | | |  |  |  |  |
| Poorest (Q1) | | | ref |  | ref |  |
| Poorer (Q2) | | | 0.38 (0.23-0.65) | <0.001 | 0.99 (0.53-1.84) | 0.966 |
| Middle (Q3) | | | 0.36 (0.21-0.60) | <0.001 | 0.64 (0.33-1.25) | 0.193 |
| Richer (Q4) | | | 0.44 (0.26-0.73) | 0.002 | 0.32 (0.15-0.69) | 0.004 |
| Richest (Q5) | | | 0.60 (0.38-0.95) | 0.028 | 0.36 (0.18-0.76) | 0.007 |
| Number of children | | |  |  |  |  |
| None | | | ref |  | ref |  |
| One or more | | | 0.66 (0.46-0.95) | 0.027 | 1.68 (1.07-2.65) | 0.026 |
| Number of residents aged 60+ | | |  |  |  |  |
| None | | | ref |  | ref |  |
| One or more | | | 0.86 (0.61-1.22) | 0.404 | 1.82 (1.15-2.89) | 0.011 |
| Outpatient care provider |  | |  |  |  |  |
| Public | | | ref |  | ref |  |
| Private | | | 3.28 (2.08-5.18) | <0.001 | 2.48 (1.41-4.35) | 0.002 |
| Not using outpatient care | | | 0.39 (0.23-0.67) | 0.001 | 0.16 (0.07-0.33) | <0.001 |
| Inpatient care provider (ref=public) | |  |  |  |  |  |
| Public | | | ref |  | ref |  |
| Private | | | 1.31 (0.83-2.07) | 0.253 | 2.38 (1.18-4.83) | 0.016 |
| Not using inpatient care | | | 0.17 (0.11-0.25) | <0.001 | 0.34 (0.18-0.65) | 0.001 |
| JKN, Jaminan Kesehatan Nasional; CI, confidence interval | | | | | | |

**Table A5. Pooled data analysis predicting the probability of incurring catastrophic health spending at 25% threshold (N=13,997)**

| **Variables** | **Odds ratio (95% CI)** | **P-value** | **Odds ratio (95% CI)** | **P-value** |
| --- | --- | --- | --- | --- |
| Health insurance coverage (ref=no coverage) | |  |  |  |
| No coverage | ref |  |  |  |
| Public (JKN) | 0.62 (0.46-0.83) | 0.001 | 0.60 (0.42-0.85) | 0.004 |
| Private | 0.72 (0.26-1.99) | 0.525 | 0.32 (0.07-1.44) | 0.136 |
| Location |  |  |  |  |
| Rural | ref |  | ref |  |
| Urban | 0.62 (0.47-0.80) | <0.001 | 0.62 (0.47-0.80) | <0.001 |
| Employment of the head of household | |  |  |  |
| Unemployment | ref |  | ref |  |
| Civil servant | 0.94 (0.53-1.66) | 0.826 | 0.95 (0.54-1.68) | 0.853 |
| Private employee | 0.52 (0.31-0.89) | 0.018 | 0.52 (0.31-0.89) | 0.017 |
| Self-employed | 0.48 (0.33-0.70) | <0.001 | 0.47 (0.33-0.69) | <0.001 |
| Household income quintile | |  |  |  |
| Poorest (Q1) | ref |  | ref |  |
| Poorer (Q2) | 0.57 (0.38-0.85) | 0.006 | 0.56 (0.38-0.84) | 0.005 |
| Middle (Q3) | 0.43 (0.28-0.66) | <0.001 | 0.44 (0.29-0.67) | <0.001 |
| Richer (Q4) | 0.40 (0.26-0.61) | <0.001 | 0.40 (0.26-0.62) | <0.001 |
| Richest (Q5) | 0.51 (0.34-0.76) | 0.001 | 0.51 (0.35-0.77) | 0.001 |
| Number of children |  |  |  |  |
| None | ref |  | ref |  |
| One or more children | 0.91 (0.68-1.22) | 0.533 | 0.91 (0.69-1.22) | 0.535 |
| Number of residents aged 60+ |  |  |  |  |
| None | ref |  | ref |  |
| One or more | 1.11 (0.84-1.47) | 0.470 | 1.11 (0.84-1.47) | 0.454 |
| Outpatient care provider |  |  |  |  |
| Public | ref |  | ref |  |
| Private | 2.93 (2.04-4.22) | <0.001 | 2.93 (2.04-4.21) | <0.001 |
| Not using outpatient care | 0.27 (0.17-0.42) | <0.001 | 0.27 (0.17-0.42) | <0.001 |
| Inpatient care provider |  |  |  |  |
| Public | ref |  | ref |  |
| Private | 1.55 (1.05-2.28) | 0.028 | 1.55 (1.05-2.29) | 0.026 |
| Not using inpatient care | 0.20 (0.14-0.29) | <0.001 | 0.20 (0.14-0.29) | <0.001 |
| Survey round |  |  |  |  |
| First wave | ref |  |  |  |
| Second wave | 0.67 (0.51-0.89) | 0.005 | 0.60 (0.37-0.98)^a^ | 0.042 |
| Insurance type # survey round |  |  |  |  |
| No coverage # first wave |  |  | ref |  |
| JKN # second wave |  |  | 1.12 (0.62-2.02)^b^ | 0.696 |
| Private # second wave |  |  | 8.09 (1.06-61.88)^c^ | 0.044 |
| JKN, Jaminan Kesehatan Nasional; CI, confidence interval; ref, reference group  ^a^ comparison is made between households with no health insurance in the second wave and households with no health insurance in the first wave (reference)  ^b^ comparison is made between households with JKN in the second wave and households with no health insurance in the first wave (reference)  ^c^ comparison is made between households with private health insurance in the second wave and households with no health insurance in the first wave (reference) | | | | |

**Table A6. A comparison of questions used to calculate out-of-pocket health payments in ENHANCE survey and SUSENAS 2017**

| **ENHANCE survey** | | **SUSENAS 2017** | |
| --- | --- | --- | --- |
| Type of visit | Item | Item number | Item |
| Outpatient | Consultation | 287 | government hospital |
|  | Administrative | 288 | private hospital |
|  | Medicine/Lab | 289 | puskesmas / pustu / polindes / posyandu |
|  | Transportation | 290 | doctor/ polyclinic practice |
|  | Informal payments | 291 | health worker practice (midwife/ nurse health order) |
|  | Food | 292 | traditional medicine practices |
| Inpatient | Consultation | 293 | birth attendant shamans |
|  | Administrative | 294 | drugs purchased by prescription from health workers (doctors, midwives, nurses) |
|  | Medicine/Lab | 295 | modern medicines that are purchased without a prescription from a healthcare provider |
|  | Transportation | 296 | traditional medicine/ herbal medicine for treatment |
|  | Informal payments | 297 | the cost of maintaining glasses, prosthetic limbs and wheelchairs |
|  | Food | 298 | check pregnancy |
|  | Room | 299 | immunization, |
|  |  | 300 | health tests/ early detection/ medical check-up |
|  |  | 301 | family planning |
